# Paramedics working in a prison-based healthcare setting: an exploratory mixed methods study

**DOI:** 10.29045/14784726.2020.12.4.4.1

**Published:** 2020-03-01

**Authors:** Lewis Johnson

**Affiliations:** Vale of York CCG NHS Trust: ORCID iD: https://orcid.org/0000-0003-1745-6824

**Keywords:** healthcare, paramedic, prison

## Abstract

**Background:**

Prison healthcare departments recently started recruiting paramedics to assist in dealing with a rise in medical emergencies largely attributed to an aging prison population and an increase in novel psychoactive substance misuse. There has been little research investigating the paramedic role in this setting. This study aims to explore the strengths and limitations of employing paramedics within the prison healthcare setting from the perspectives of non-paramedic colleagues.

**Methods:**

An exploratory mixed methods study was conducted in a UK category B remand prison, focusing on the opinions and observations of current healthcare and custodial staff. Paper questionnaires were completed by 32 members of staff and semi-structured interviews were conducted with two participants.

**Results:**

Seven global themes were identified within the qualitative data: management of medical responses; effect of a specialist role; effect on ambulance escorts; contribution to professionalism within the department; effect on the role of other healthcare staff; prisoner interaction with paramedics; and difficulties encountered in role implementation. Of the 32 participants, 31 believe paramedics have had an overall positive effect on the provision of healthcare, with a variety of reasons explored.

**Conclusion:**

In a small exploratory study, it is suggested that paramedics possess the relevant skills and training to offer a meaningful contribution to the provision of prison healthcare; however, further research is required to explore the full scope of their contribution in this setting.

## Introduction

UK prison populations have risen by approximately 66% since 1995, with 80/116 prisons in England and Wales currently exceeding their certified normal accommodation. This overcrowding has been attributed to an increased incidence of assaults and self-harm among prisoners (Allen & Watson, 2017). In addition to overcrowding, a rapid rise in the use of novel psychoactive substances (NPSs) has shown a 397% increase in the number of deaths associated with NPSs in the period between 2011 and 2016 (HM Inspectorate of Probation, 2017). The average age of prisoners has been steadily increasing, with the 50+ age group having disproportionately risen by 169% since 2002. This has contributed to 2016 recording a record high rate of deaths per prisoner due to natural causes (2.3/1000), and a record rate of total deaths in custody (4.15/1000) (Allen & Watson, 2017). With the increase in demand and complex care needs of prisoners, the delivery of safe and effective healthcare within this setting presents a unique and increasingly difficult challenge for healthcare professionals, often requiring nursing staff to work ‘beyond their training and competency’ in order to meet the increasing and complex demand (Condon, Gill, & Harris, 2007). Recruitment and retention of GPs and general nurses has presented as particularly difficult in the prison setting. This can result in a lack of GP and nurse support available to prisoners using the traditional appointment structure (Cornford et al., 2008), causing departments to adapt and consider alternative means of meeting demand.

With increasing exposure to higher education and advanced clinical assessment skills, paramedic practice has moved away from the model of a simple patient transport service and adopted a more integrated role within the NHS, branching out into a variety of primary care roles such as in general practice, accident & emergency and walk-in centres (Ball, 2005), often autonomously reducing the rate of emergency department conveyance (Evans, McGovern, Birch, & Newbury-Birch, 2014). Until recently the majority of paramedic employments within offender health were in police custody, with only sporadic positions occupied within prison healthcare. March 2017 saw the introduction of 24-hour paramedic cover within a UK prison at HMP/YOI Doncaster, a male category B remand facility with a capacity of 1145. Currently no published studies have explored the strengths and limitations of the paramedic role within a prison setting.

This study aimed to explore the strengths and limitations of the paramedic role within this prison environment. This was achieved by focusing on three questions: (1) How has the introduction of paramedics into the prison-based healthcare setting been perceived by prison officers and other healthcare staff? (2) Have other staff at the prison observed a change in practice within the prison since the employment of paramedics? (3) Since employing paramedics, has patient care changed when responding to a ‘code red/blue’ emergency?

## Methods

### Study population

This study took place between November 2017 and May 2018. Clinical healthcare staff excluding paramedics (36) and operational custodial staff (260) employed at HMP/YOI Doncaster within this time period were eligible to take part in this study. Although the opinions and experiences of the paramedic group involved are relevant to this study, their inclusion could have introduced a conflict of interest. Therefore this study focuses on the experiences and perceptions of non-paramedic colleagues, and the five paramedics involved were excluded from the sample population to remove a source of bias from the data.

### Recruitment

Convenience sampling dependent on availability and shift patterns coinciding with the researcher was undertaken for questionnaire distribution and collection purposes. Participation was voluntary and participants were offered an incentive of entry into a draw to win vouchers of monetary value via a questionnaire attachment. All participants were employed at HMP/YOI Doncaster and had varying experiences, as shown in Table 1. A single questionnaire was permitted for each participant and was recorded on return. Data saturation was considered unlikely due to the large sample population, and no limit was placed on the sample group size.

**Table 1.** Demographic of participant role and experience working in a prison environment.

	Number (N)	Mean number of years at HMP Doncaster (range)	Mean number of years working in a prison environment (range)
**Healthcare** Registered general nurse Substance misuse nurse Healthcare assistant Role unknown	9 3 1 2 3	6.94 (0.33–16.00) 2.00 (0.33–4.75) 5.50 (5.50–5.50) 13.21 (10.42–16.00) 3.94 (1.67–11.83)	7.46 (0.33–16.00) 2.08 (0.33–5.00) 10.00 (10.00–10.00) 13.21 (10.42–16.00) 3.94 (1.67–11.83)
**Custody** Operational manager Prison custody officer Operational support officer Role unknown	23 2 5 1 15	7.77 (0.17–23.67) 13.13 (2.75–23.50) 7.03 (2.00–23.50) 0.17 (0.17–0.17) 7.81 (0.25–23.67)	9.30 (0.17–30.75) 21.75 (20.00–23.50) 9.23 (2.17–23.50) 0.17 (0.17–0.17) 8.28 (0.25–30.75)

Within the questionnaire, participants were asked if they would consent to a further interview. Following data analysis of the questionnaire, interview volunteers were anonymously compared and selected for invitation using a purposive sampling technique corresponding to the number of global themes discussed in their questionnaire. The volunteer who discussed the broadest range of identified themes was selected from each of the healthcare and custody groups, both of whom accepted.

### Study design/data collection

To explore the paramedic role in this environment, a predominantly qualitative mixed methods study was conducted with staff in the prison. Phase one involved completion of an anonymous paper questionnaire consisting of open questions and categorical multiple-choice questions regarding the participants’ observations of paramedic practice (Supplementary 1). This was distributed to staff during shifts within the prison for completion at their convenience. Content was chosen based on the observations of the paramedic researcher within the department over nine months prior to the study. This was followed by face-to-face semi-structured interviews with two participants designed to contribute further depth to the recurrent global themes previously identified in the questionnaire. These were conducted one-to-one in private rooms within the prison at the convenience of both parties; a prompt sheet was utilised and audio recordings made for later analysis. Interviewees were aware of the study aims.

### Data analysis

**Figure 1:**
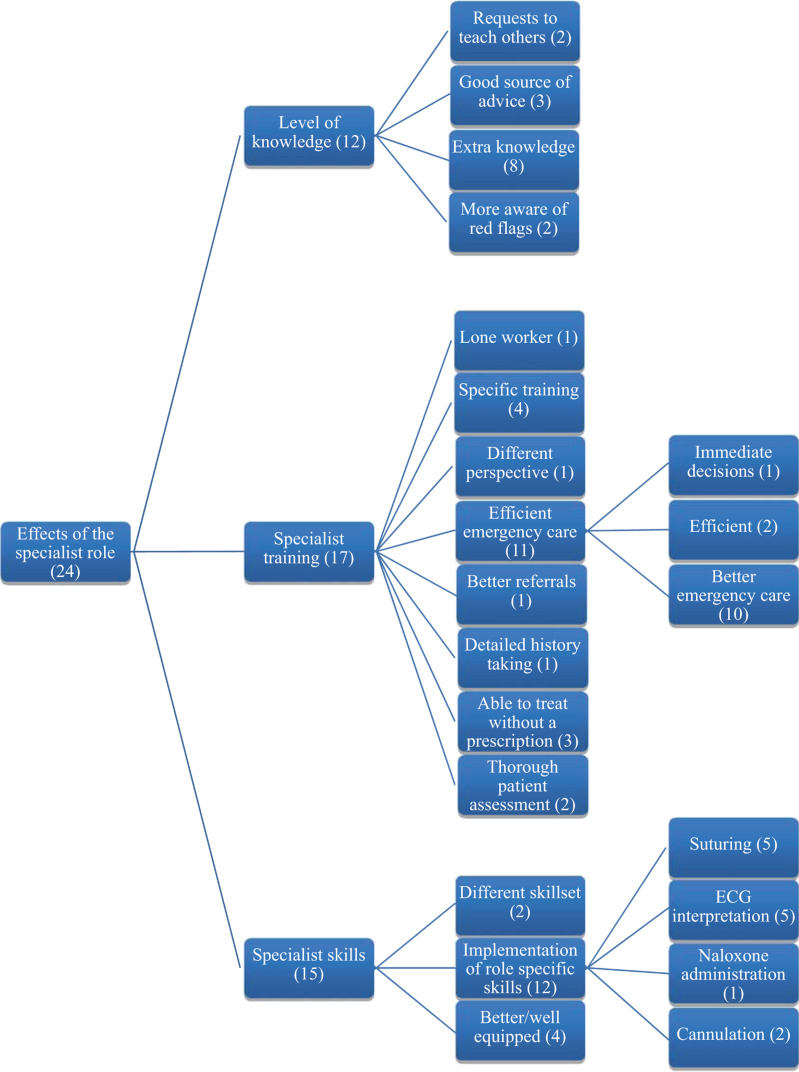
Figure 1. Network analysis demonstrating the coding leading to the global theme of the ‘effects of the specialist role’.

Note: Bracketed numbers indicate the number of individual participants contributing to the code/theme.

**Figure 2:**
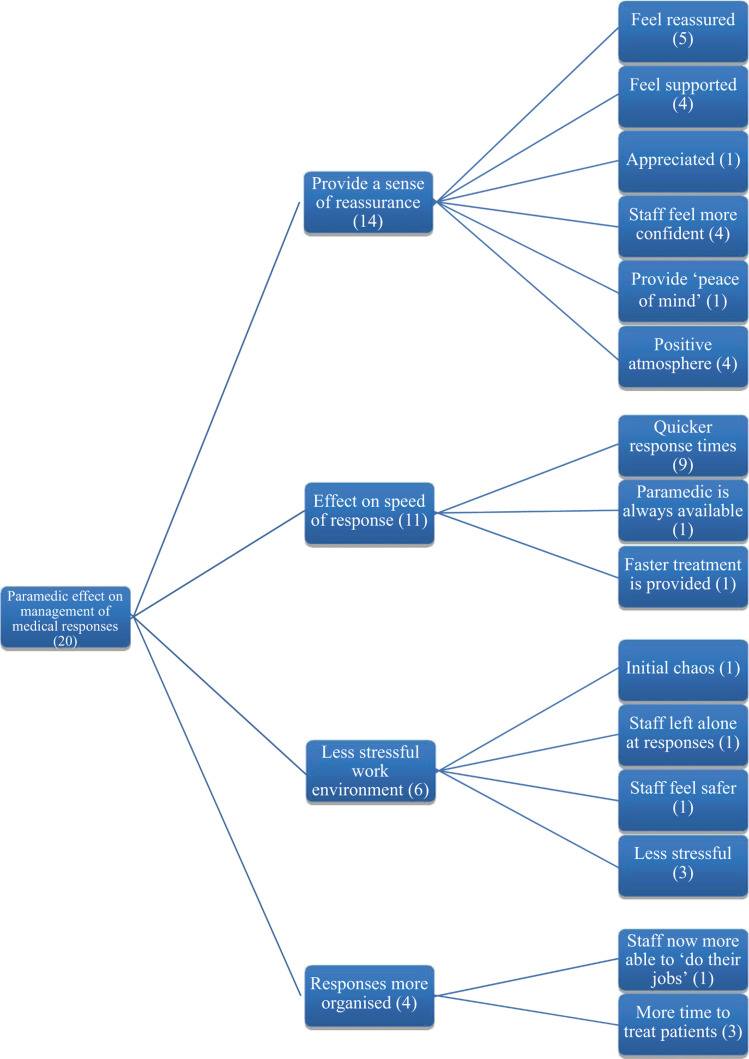
Figure 2. Network analysis demonstrating the coding leading to the global theme of the ‘paramedic effect on management of medical responses’.

Note: Bracketed numbers indicate the number of individual participants contributing to the code/theme.

Analysis of categorical quantitative questionnaire data consisted of simple correlation and representation using descriptive statistics to illustrate the general opinions of the sample population. Thematic network analysis was carried out on the qualitative questionnaire data by the researcher using a tabletop method as described by Creswell and Plano Clark (2011). This consisted of extracting identified codes to paper and correlating them into basic themes before developing them into organised and global themes respectively, as shown in Figures 1 and 2. An initial 81 codes were reduced to seven global themes recurring throughout the study through a process of gradually grouping comparable codes into developed themes and regrouping developed themes under blanket global theme headings. These global themes were subsequently explored during the interviews which were transcribed and analysed using a line-by-line approach to identify and code each individual remark to its relevant theme. Any content considered to be insightful by the researcher was highlighted and extracted according to its significance towards contributing depth to the global theme. Interview durations ranged from 15 to 25 minutes, and no pilot or repeated interviews were carried out.

## Results

Nine of the eligible 36 healthcare staff (25%) and 23 of the eligible 260 custodial staff (8.8%) took part in the study. Thirty of the 32 (94%) participants asked agreed that overall paramedic introduction has had a positive effect on the provision of healthcare in the prison. Twenty-nine (91%) agreed it had made healthcare more accessible to prisoners, largely due to their increased availability to travel around the prison. Twenty-seven (84%) agreed the quality of non-emergency care available to prisoners had increased. Twenty-seven (84%) and 28 (88%) agreed paramedic introduction had improved the management of code blue and code red medical responses respectively. There was no clear trend towards paramedics’ effect on transport out to secondary hospital care. None of the quantitative data collected were suggestive of paramedics working in the prison being detrimental to practice (Table 2).

**Table 2.** Quantitative representation of participants’ opinions on how paramedics have affected overall practice.

	Strongly disagree	Disagree	Agree	Strongly agree	Do not know
Paramedic employment has had a positive effect on the prison’s overall provision of healthcare.	0 (0.0%)	1 (3.1%)	12 (37.5%)	18 (56.3%)	1 (3.1%)
The paramedic triage service clinic has made healthcare more accessible for prisoners.	0 (0.0%)	0 (0.0%)	13 (40.6%)	16 (50.0%)	3 (9.4%)
The paramedic triage clinic/service has increased the quality of non-emergency care available to prisoners.	0 (0.0%)	2 (6.3%)	13 (40.6%)	14 (43.8%)	3 (9.4%)
Paramedics have had a positive effect on the management of code blue emergency responses.	0 (0.0%)	1 (3.1%)	8 (25.0%)	19 (59.4%)	4 (12.5%)
Paramedics have had a positive effect on the management of code red emergency responses.	0 (0.0%)	1 (3.1%)	8 (25.0%)	20 (62.5%)	3 (9.4%)
Paramedics are more likely to send a prisoner out to hospital for further assessment.	2 (6.3%)	15 (46.9%)	7 (21.9%)	0 (0.0%)	8 (25.0%)

Twenty-three (72%) participants agreed the treatment of prisoners experiencing chest pain, NPS/spice incidents and assaults had improved with paramedic introduction. Twenty-four (75%) agreed treatment of breathing difficulties had improved, and 20 (63%) agreed treatment of self-harm had improved. There was no clear trend towards paramedics’ effect on treatment of prisoners experiencing a mental health crisis. None of the included presenting complaints suggested a reduced quality of care with paramedic introduction (Table 3).

**Table 3.** Quantitative representation of participants’ opinions on how paramedics have affected the treatment of individual presenting complaints.

	Much worse	Worse	Unchanged	Better	Much better	Do not know
Chest pain	1 (3.1%)	0 (0.0%)	3 (9.4%)	9 (28.1%)	14 (43.8%)	5 (15.6%)
Breathing difficulty	1 (3.1%)	1 (3.1%)	1 (3.1%)	13 (40.6%)	11 (34.4%)	5 (15.6%)
NPS/spice attack	0 (0.0%)	0 (0.0%)	6 (18.8%)	7 (21.9%)	16 (50.0%)	3 (9.4%)
Assaults	0 (0.0%)	1 (3.1%)	5 (15.6%)	12 (37.5%)	11 (34.4%)	3 (9.4%)
Self-harm	0 (0.0%)	0 (0.0%)	9 (28.1%)	11 (34.4%)	9 (28.1%)	3 (9.4%)
Mental health crisis	0 (0.0%)	2 (6.3%)	17 (53.1%)	5 (15.6%)	2 (6.3%)	6 (18.8%)

Note: NPS = Novel Psychoactive Substance.

Seven global themes were recurrent throughout the questionnaire and are outlined below. Quotes extracted from interviews are identified; all others are extracted from anonymous questionnaire open questions.

### 1. Effect of a specialist role

The broadest global theme identified can be categorised under the effect of implementing a new specialist role, which is shown in Figure 1. This consisted of observations of the knowledge, training and skills specific to paramedics and the effect these have had on patient care in the prison. Participants agreed that the treatment of patients experiencing chest pain had been improved by the availability of paramedics, as shown in Table 3. This was largely due to experience and training in interpreting electrocardiograms (ECGs) which previously could only be done autonomously by an on-site GP. Table 3 also shows that participants agreed that the treatment of patients with breathing difficulties had been improved by paramedics. Healthcare staff suggested that this was largely due to the treatment of acute breathing problems and a paramedic’s autonomy in administration of oxygen driven nebulisers under a patient group direction without the need for a prescription. A common impression is that paramedics provided an additional dimension to patient care by contributing a ‘different perspective’. This has resulted in the implementation of emergency care practices that were previously not available to healthcare staff such as use of nasopharyngeal airways, availability of Entonox and intravenous infusion of fluid and medication. A member of the nursing staff explains:

We can give IV fluid now, and that’s something that they’ve [paramedics] introduced. But at one time I shared their frustration, we were really frustrated that we couldn’t do that, that we’d never even tried to move it forward. You know there were nurses here who could actually do that, but that’s a really good example of looking at something, a paramedic looking at it differently, taking it by the horns and making the change. (Healthcare interview)

Twelve participants contributed complimentary remarks regarding the depth of specialist clinical knowledge possessed by paramedics, with three healthcare staff stating that they provided a ‘good source of advice’. It was suggested by an individual that a wider diversity in knowledge and training across the department may have contributed a positive effect on patient safety, stating that:

they [paramedics] may have picked up on something that the other nurses may not have done that might save the prisoner’s life by sending them out [to hospital]. (Custodial interview)

### 2. Management of medical responses

One of the primary duties of the paramedic role is to attend and manage both ‘code red’ and ‘code blue’ medical responses. A code blue is defined as a potentially life-threatening medical emergency as triaged by a non-clinical member of staff, where the patient is presenting as unconscious, experiencing chest pain or shortness of breath, commonly attributed to NPS/substance misuse. A code red is defined as a medical emergency where the patient is experiencing significant bleeding, usually caused by assault or self-harm. Overall, 27 and 28 of the sample population felt paramedics had improved the management of code blue and code red medical responses respectively, as shown in Table 2, and 23 felt the treatment of NPS/spice incidents and assaults had been improved by the introduction of paramedics, as shown in Table 3. The most frequently mentioned aspect of paramedic management of medical responses was an increased speed of arrival on scene, as illustrated in Figure 2. This observation generated common remarks from custodial staff such as, ‘[I’m] reassured that someone will be on scene more promptly’, and ‘They generally can be a quicker response than waiting for a nurse, when the nurses are occupied with other things’. Previously, nursing staff were required to attend medical responses, which involved interruption of regimented medication rounds that could not always be suspended quickly; paramedics were not assigned medication duties so were more readily available to attend medical responses. The impression that response times were shorter was often accompanied by feelings of reassurance, support and confidence, primarily from the custody staff who declared the medical response. ‘Staff appear to be calmer because they know a paramedic will arrive shortly after the alarm is raised’ was a common observation. This is supported by references from both healthcare and custodial staff to a previously stressful working environment during a medical response, with one description of an ‘initial chaos’ when a code blue was declared and others simply stating they now feel less stressed when declaring a medical response when a paramedic is on duty.

### 3. Effect on ambulance escorts to hospital

Transporting prisoners to hospital for treatment is a costly burden for the prison service and requires two to three staff members per prisoner to be reallocated away from the prison to provide hospital escort cover. Table 2 shows that 17 participants thought paramedics were no more likely to send a patient to hospital for treatment than any other members of healthcare staff. Nine individuals suggested ambulance calls had been reduced within the open questions, with none suggesting an increase, as illustrated in Figure 2. One interview participant summarised:

The paramedic may be able to deal with something on site rather than send out a prisoner, this has a massive effect on the prisoner, saving key resources and keeping officers in the prison. (Custodial interview)

Participants suggested the management of patients under the influence of NPSs has improved with the introduction of paramedics, as shown in Table 3. This was previously suspected to be one of the highest causes of unnecessary hospital admissions and it is suggested that a change in the management and decision making around this patient group has contributed to some participants reporting the observation of fewer hospital admissions. A nurse highlighted the importance of providing care within the prison setting:

The paramedic’s role is a bit like a gatekeeping role in a lot of cases. To not send people out unnecessarily because you have to consider the increased risk to the patient, to the staff, to members of the public. (Healthcare interview)

### 4. Contribution to professionalism

Prisoners can often present as volatile and confrontational towards healthcare staff, and professionalism is an important attribute to demonstrate across the department. A high level of professionalism among paramedics was frequently observed within the sample group, with references to documentation, attitude and inter-professional communication demonstrating effective team working and leadership. Other common observations were of a calm and confident influence contributing to a generally more positive atmosphere and reassurance from colleagues, summarised by the statement, ‘[Paramedics are] very professional and calm in their approach and dealt easily with any situation’.

### 5. Effect on roles of other healthcare staff

The paramedic’s primary duties were to attend medical responses and manage urgent primary care issues which were previously the responsibility of nurses. The feedback was mixed regarding paramedic influence on a nurse’s level of responsibility within the department. One common view was that having a paramedic present to deal with medical responses alleviates pressure on nurses and reduces the disruption caused during medication time. This generated statements such as, ‘I am able to concentrate on my own duties as a nurse with no interruption from responses’. The contrasting view presented was that because paramedics were not assigned duties such as dispensing medications and conducting initial reception screens, this increased pressure on nurses to compensate and fulfil these duties.

### 6. Prisoner interaction with paramedics

There was a strong suggestion that prisoners react positively towards paramedics, with 13 participants mentioning this observation. A show of respect towards the paramedics was a common theme, with some staff suggesting prisoners felt ‘looked after’ and were ‘more relaxed and less confrontational towards paramedics’. The only negative observation was of prisoners attempting to manipulate the availability of paramedics to gain something from custodial staff, such as time out of their cell while on basic regime.

### 7. Difficulties encountered

There was a suggestion that healthcare demand may have increased due to the availability of paramedics, with prisoners more likely to report complaints due to paramedics’ increased tendency to attend prison wings to carry out assessments instead of in the healthcare department. Observations of inappropriate referrals to paramedics and inefficient utilisation of the role were the most common difficulties highlighted. ‘They [paramedics] are overused and not always available due to staffing limitations’ was one statement, while another observed a reason for this was due to prisoner preference moving paramedic demand away from emergency care: ‘Prisoners now ask for paramedics at times for trivial problems’.

## Discussion

This study provides an exploratory insight into the observations of current prison staff regarding the introduction of paramedics into a prison healthcare setting, and is not intended to provide definitive conclusions regarding the role. The findings highlight the complexities of implementing a new role into an established healthcare system and illustrate the strengths and limitations observed by those in regular contact with the paramedics. This is the first time a study of this type has been reported.

The utilisation of paramedics as a dedicated emergency responder is perceived by custody staff to have reduced the response time for medical emergencies throughout the prison, improving clinical governance by increasing the quality of care provided and reducing the risk to patients. It is suggested that the increased availability of nebulisers, analgesia, intravenous therapy and ECG interpretation provided by paramedic employment has increased the quality and range of patient care available within the prison. Both custodial and healthcare staff appear comfortable seeking clinical advice from a paramedic, contributing to an overall increase in confidence and support when making decisions regarding patient care. The extension to the clinical experience and training within the department provided by paramedics, predominantly in the provision of primary and emergency care, appears to offer a sense of support and reassurance to other healthcare staff who would previously have been required to work beyond their levels of training and competence (Condon, Gill, & Harris, 2007) to meet the increasing demand (Allen & Watson, 2017; Kuzak et al., 2001). The number of patients requiring advanced assessment for antibiotics, analgesia and drug and alcohol detoxes in the prison setting is high, and there remains potential to expand the paramedic role in a prison setting to include prescribing, and further reduce demand on these primary care services.

References from participants to a high level of specialist knowledge particularly regarding ECG interpretation, acute wound care and referral procedures have resulted in a perceived reduction in the number of prisoners requiring transportation to hospital for treatment, although this finding is not definitive and requires further investigation. These specialisms in conjunction with enquiries to teach other members of staff suggest paramedics may be able to contribute to the development of an educational system within offender health, and assist integration between the prison healthcare services and other healthcare departments in the community, as recommended by Watson, Stimpson, and Hostick (2004).

Paramedic employment may present as a means of counteracting the ongoing difficulties in recruiting and retaining prison nursing staff, as discussed by Cornford et al. (2008), Chafin and Biddle (2013) and Wraith (2017). The difference in training and skills between the professions and the high demand for traditionally nurse specific duties, such as the daily dispensing of prescribed medication and provision of social care for the elderly, suggest that without extending paramedic practice to include a wider variety of nursing skills, paramedics are not a suitable like-for-like replacement for nurses but rather a supplementary addition to a multidisciplinary healthcare team. Further investigation would be required to explore the effect this would have on the current strengths of the role and the desirability from a paramedic recruitment and retention viewpoint. It is worth noting that 18 months following the data collection for this study, none of the five paramedics involved remain working at the prison, suggesting difficulties retaining staff may not be specific to the nursing profession but with healthcare staff in general.

### Limitations

The current dearth of research regarding paramedics in a prison setting means the findings of this study lack support from a variety of methods and sources. This study was conducted by a member of the paramedic team being explored in the study, presenting a conflict of interest, and the decision to exclude paramedics from the sample population was an attempt to reduce bias for this reason; however, the absence of data from this significant group can also be viewed as a limitation and would need to be investigated in a future study. The sample size consisted of 10.8% of the sample population, resulting in a quantitative margin of error at 16.4%. Although this is large enough to represent a useful insight as intended in this study, a greater sample size would offer additional reliability. Although often associated with increased bias and sampling error, a convenience sampling technique was chosen as a strategy to maximise participation. A stratified random sampling technique would be preferable in a larger study with larger sample size. The paramedic interviewer and interviewees were professionally familiar due to mutual employment within the facility; this is likely to have presented a positive bias towards the feedback given; however, the interviews were intended only as supplementary insight into previously identified themes and not independently from the quantitative data. Although efforts were made to minimise bias during sampling and data collection, data analysis and interpretation of results were conducted by an individual researcher and wider contribution may have offered additional insight. Transferability of the findings is limited to comparable category B remand prisons with a similar infrastructure and prisoner and staff demographic.

### Future research

This study provides a basic insight into the effects a team of paramedics have had on one prison and provides a foundation for future research. Escorting prisoners to hospital for treatment contributes significant cost to the prison service and exploring methods of providing a wider range of treatments within the prison setting is of benefit to both the prisons financially and patients clinically. There remains an opportunity for a quantitative study to investigate the effect paramedic availability can have on the frequency and types of patients being sent out to hospital for treatment, and the financial implications involved. There remains a variety of avenues paramedic practice can pursue to further integrate into offender health. Future studies may explore the effects of expanding paramedic practice within offender health to include independent prescribing, medicine management or other secondary care services.

## Conclusion

This small-scale exploratory study suggests that although they cannot be considered a replacement to current nursing staff working in the prison setting, paramedics possess the relevant skills and training to offer a meaningful contribution to the provision of prison healthcare. However, further research is required to explore the full scope and limitations of paramedics in a prison healthcare setting.

## Acknowledgements

This study could not have been undertaken without the support and participation of staff at HMP/YOI Doncaster, and the mentorship and support of Professor Julia Williams at University of Hertfordshire, throughout the research process.

## Conflict of interest

None declared.

## Ethics

The study received ethics approval from the University of Hertfordshire Health, Science, Engineering and Technology Ethics Committee with Delegated Authority. Consent was implied on completion and return of the questionnaire, and care was taken to protect participant anonymity throughout. Prior to undertaking interviews, volunteers were provided with an information sheet and informed of the purpose of the study before signing a consent form, reserving the right to withdraw at any time.

## Funding

None.
